# Gender differences in functional disability and self-care among seniors in Bangladesh

**DOI:** 10.1186/s12877-017-0577-2

**Published:** 2017-08-08

**Authors:** Md. Ismail Tareque, Andrew D. Tiedt, Towfiqua Mahfuza Islam, Sharifa Begum, Yasuhiko Saito

**Affiliations:** 1 Department of Population Science and Human Resource Development, University of Rajshahi, Rajshahi, Bangladesh; 2 United States Department of Justice, Washington DC, USA; 3 Bangladesh Initiative for Sustainable Future (BiSF), Dhaka, Bangladesh; 4 Population Studies Division, Bangladesh Institute of Development Studies (BIDS), Dhaka, Bangladesh; 5 University Research Center and School of Medicine, Nihon University, Tokyo, Japan; 6 Duke-NUS Graduate Medical School, Singapore, Singapore

**Keywords:** Disability, Old age, Gender disparities, Low income country, Bangladesh

## Abstract

**Background:**

Disability among older adults is a public health concern. To date there are no in-depth and comprehensive analyses on older adults’ disabilities in Bangladesh. This study investigated gender differences in the prevalence of disability and the socio-demographic factors associated with disability among older adults in Bangladesh.

**Methods:**

This research used a sample of 4176 elderly males and females aged 60 years and over from a nationally representative data set- Bangladesh’s 2010 Household Income and Expenditure Survey. The study used both household level and individual level data and applied a wealth index, which was constructed based on household assets using principal component analysis. The Washington Group’s short set of questions on disability were used to measure disability. Chi-square tests and ordinal logistic regression models were fit.

**Results:**

Forty-two percent of older had some form of functional disability, including 5% of elderly with severe/extreme functional disability. Seven percent of older adults had a self-care disability, including 3% of elderly with a severe/extreme form of self-care disability. Elderly females suffered from all the studied disabilities, including functional and self-care disabilities in higher percentages, and had higher odds ratios of having both functional disability and self-care disability compared to elderly males. The study also identified some significant factors affecting functional disability and self-care disability, namely age, having a chronic condition, wealth status and place of residence, including divisional differences.

**Conclusions:**

Programs aimed at reducing functional disability among seniors, particularly elderly females, should be granted the highest priority in Bangladesh.

**Electronic supplementary material:**

The online version of this article (doi:10.1186/s12877-017-0577-2) contains supplementary material, which is available to authorized users.

## Background

Population aging is occurring rapidly across the globe and is evident in less wealthy nations such as Bangladesh. A large body of research has investigated problems associated with aging in the developed world, but there have been few studies on Bangladeshi older adults. Since the less developed nations are currently aging at a faster rate than countries of the developed world [1], due attention should be placed on elderly-oriented research in Bangladesh.

There is a conspicuous lack of disability research among studies focused on aging in Bangladesh and throughout much of the less-developed world. Disability may result from an event at or prior to birth; it may occur as a result of acute onset of disease, injury or chronic conditions that over time affect physical or cognitive function in mid to later life [2]. For some people it represents a lifelong experience, whereas for others it is a late-life concern. In old age, people have impaired functioning due to progressive loss of physiological integrity in the aging process. Functional decline, a form of disability, in old age results not only from acute diseases but also from frailty (weakness due to aging) [3]. Functional disability is common in older adults, and it is often episodic and is associated with a high risk of subsequent health decline [4]. Mobility limitation, a one-leg balance stand less than five seconds or walking speed less than 0.8 m per second, predict future disability among older people [5]. An unhealthy lifestyle, characterized by physical inactivity, an unhealthy diet, and smoking, are also reported to be associated with greater hazard of disability among older people [6]. However, a structured, moderate-intensity physical activity program can reduce major mobility disability among older adults [7]. Disability can thus be considered a significant issue and public health concern. It is associated with poverty and quality of life.

Bangladesh, a South Asian nation, has made substantial progress in reducing poverty and has achieved the central United Nations Millennium Development Goal of halving poverty ahead of the target deadline. Rapid economic growth enabled Bangladesh to reach the status of a lower middle-income country in 2014. Despite improvements in poverty reduction, food security, education for girls, and creation of economic opportunities for women and women’s political participation, Bangladesh scored poorly on gender indices. It was ranked 111th in the Gender Inequality Index and 142nd in the Human Development Index in 2014 [8]. The population of Bangladesh was 158.1 million with a population density of 1077 per square kilometer in 2015 [9]. Around 66% of citizens live in rural areas, and the rest in urban areas. There were seven divisions (Barisal, Chittagong, Dhaka, Khulna, Rajshahi, Rangpur, and Sylhet) that divide Bangladesh into seven major administrative regions until 2014. Each division is named after the major city (within the division’s jurisdiction) that serves as the administrative capital of that division. Since 2015, Bangladesh has been divided into eight divisions; the newly added Mymensingh division was created from districts previously comprising the northern part of the Dhaka division. There are different estimates of disability prevalence reported in Bangladesh, arising from distinct survey instruments. Tareque and colleagues [10] defined ‘activities in daily living limitations’ as an inability to perform usual daily activities such as eating, dressing, and bathing and ‘physical limitations’ as difficulty squatting, lifting objects weighing 5 kg or more, walking about 1 km, and climbing stairs (2–3 steps). They reported that the prevalence of activities in daily living limitations was 2.7% and the prevalence of physical limitations was 42.6% among the elderly in the Rajshahi district of Bangladesh. Using data from Bangladesh’s Household Income and Expenditure Survey (HIES)-2010, Tareque and colleagues [11] found that 39% of males and 46% of females aged 60 years and over had a disability. The HIES adopted the Washington Group’s (WG) short-set of questions on disability. Tareque and colleagues [11] created a cut-off with 2 groups from 4 response categories of the WG’s six questions on disability as “yes (yes, some difficulty / yes, severe difficulty / yes, can't see/hear/walk/remember/self-care/communicate at all)” and “no (no difficulty)” for conceptualizing disability as having at least one among the six limitations (vision, hearing, walking and climbing, remembering and concentrating, self-care, and speaking and communicating) in their study. Using data from respondents aged 5 years and over from the same HIES-2010, Tareque and colleagues [12] used the same cut-off as the previously mentioned study [11] and reported that 9% of males and 11% of females in Bangladesh had a disability. Using the Rapid Assessment of Disability (RAD) survey conducted in the Bogra district of Bangladesh, Marella et al. [13] considered a different cut-off than Tareque et al. [11], and reported 9% of respondents aged 18 years and older had a disability. The RAD survey collected data on the prevalence of disability based on activity limitations similar to the WG questions [13]. However, disability prevalence and its severity among senior citizens has never been reported for the entirety of Bangladesh.

Research on gender differences in health and disability are of considerable interest to researchers and policy makers, and a number of studies have been conducted on these issues in different settings [11, 14–20]. Previous studies found several socio-demographic variables, including gender, that are associated with disability [11, 12, 14, 20–27]. Older adults with multiple chronic diseases, poor self-assessed health conditions, and who practice sedentary life styles had high prevalence of functional disability [28]. The study by Tareque et al. [12] found that socio-economic status as measured by a wealth index from household assets was significantly associated with disability among respondents aged 5 years and over. It also identified factors that affect disability, e.g., age, sex, marital status, place of residence including divisional differences. Older rather than younger, female rather than male, non-currently married versus currently married, people from rural areas as opposed to people from urban areas, and people from Rajshahi division rather than people from other divisions were reported to have higher disability [12]. Age, socio-economic status and employment status were likewise found to influence disability among people aged 18 years and over [13]. Compared to elderly males, among all age groups elderly females were reported to have longer life expectancy, but higher prevalence of disability and shorter disability-free life expectancy [11]. However, no study has conducted in-depth analyses on older adults’ disabilities that are representative for the population of Bangladesh. Therefore, the present study, a continuation of the study by Tareque and his colleagues [11], examined gender differences in prevalence of disability and the assessment of the factors contributing to disability among older Bangladeshi.

## Methods

### Data

This study utilized data from a large nationally representative survey, HIES-2010, collected by the Bangladesh Bureau of Statistics (BBS), an organization of Bangladesh’s Ministry of Planning. The objectives, sampling design, and methodologies of HIES-2010 were described elsewhere in detail [29]. In brief, the survey provided household level data on household income, expenditures, assets, housing conditions and individual level data on education, health, and disability. The sample of HIES-2010 was drawn employing a two-stage, stratified, random sampling design. The data were collected from February 1, 2010 to January 31, 2011. A total of 12,240 households were selected, and all individuals from selected households were interviewed for a total of 55,580 individuals. The sample for this study was restricted to those who were 60 years of age and above. The sample, thus, consisted of 4176 elderly male and female; 2145 (51.36%) were male and the rest were female.

### Measures

#### Outcome variables

We used two main outcome measures of disability, namely functional disability and self-care, generated from the short set of six disability questions developed by the Washington Group. The set of questions was consistent with the International Classification of Functioning, Disability and Health, a framework for conceptualizing disability developed by the World Health Organization [30]. The six questions were: (1) Do you have difficulty seeing, even if wearing glasses? (2) Do you have difficulty hearing, even if wearing a hearing aid? (3) Do you have difficulty walking or climbing steps? (4) Do you have difficulty remembering or concentrating? (5) Do you have difficulty with self-care such as washing all over or dressing, feeding, toileting, etc.? And (6) Do you have difficulty communicating, for example, understanding or being understood? Each question (disability) has four response categories: (a) no difficulty, (b) yes, some difficulty, (c) yes, severe difficulty, or (d) yes, can’t see/hear/walk/remember/self-care/communicate at all. Keeping the same 4-point ordinal scale as originated from the HIES-2010, each disability was categorized as: “no” (no difficulty), “moderate” (yes, some difficulty), “severe” (yes, severe difficulty), and “extreme” (yes, can’t see/hear/walk/remember/self-care/communicate at all). Five functional domains, (1) vision, (2) hearing, (3) walking and climbing, (4) remembering or concentrating, and (5) communicating were combined to generate functional disability, and were separate from self-care. Functional disability was defined as having at least one among the five functional disabilities, and was categorized into 4 groups: “no” (no disability among the five functional domains), “moderate” (any moderate disability but no severe or extreme disability among the five functional domains), “severe” (any severe disability but no extreme disability among the five functional domains), and “extreme” (any extreme disability among the five functional domains). Self-care was maintained in the original format from the HIES-2010 with the same 4-point ordinal scale of no, moderate, severe, and extreme. Additionally, a third-outcome “general disability” was generated from all the studied disabilities (six disability questions). General disability was defined as having at least one among all the studied disabilities, and was also categorized into 4 groups: “no” (no disability among all the studied disabilities), “moderate” (any moderate disability but no severe or extreme disability among all the studied disabilities), “severe” (any severe disability but no extreme disability among all the studied disabilities), and “extreme” (any extreme disability among all the studied disabilities).

#### Independent variables

The literature covered in the introduction guided the selection of variables for analyses. Following standard procedure, the socio-demographic variables available in the data set were included as independent variables in the current study. The independent variables used in this study were categorical age (expressed as groups: 60–64, 65–69, 70–74, 75–79, and 80 and over), sex (male and female), marital status (currently married and others, where others includes never married, widowed, divorced, and separated), dichotomous education (illiterate and literate), a dichotomous indicator that described suffering from at least one of ten chronic conditions (chronic heart disease, asthma/breathing trouble, chronic dysentery, gastric/ulcer, blood pressure, arthritis/rheumatism, diabetes, cancer, paralysis and epilepsy) in the 12 months preceding the survey, a wealth index (poor, middle, and rich), place of residence (rural and urban), and the Bangladesh divisions (7 divisions, namely, Barisal, Chittagong, Dhaka, Khulna, Rajshahi, Rangpur, and Sylhet). The wealth index was constructed using 27 household assets from household level data (*N* = 12,240). The procedure for generating the wealth index has been described elsewhere in detail [12]. The wealth index was created following Filmer and Pritchett’s method [31] of employing Principal Component Analysis. The wealth index values from household level data were assigned to all individuals (*N* = 55,580) based on common variables in both household and individual level data sets, and finally the individual level data were analyzed. Individuals were then ranked from top to bottom according to the index value as suggested by Rutstein and Johnson [32]. We established cut-off values for percentiles of the population, and we referred to the bottom 40% as “poor”, the next 40% as “middle”, and the top 20% as “rich” following Filmer and Pritchett’s study [31]. The same cut-offs had also been used in a disability study in Bangladesh [12].

### Statistical analysis

The analyses first examined descriptive characteristics of the study population and tested gender differences using chi-square tests. Gender differences in disability status were also assessed using chi-square tests. Then, factors associated with functional disability and self-care were examined for the male and female data sets separately and for the pooled data using ordered logistic regression models. The first set of regression models displayed crude odds ratios revealing the bivariate associations between the dependent and independent variables. However, a Brant’s test [33] found that the proportional odds assumption had been violated in the models that adjusted for socio-demographics. Although violations of proportionality are common in ordered logistic regression, since it is rare that all levels of independent variables have the same relationship with the dependent variable, we also fit separate generalized ordered logistic regression models as a check. Generalized ordered logistic regression has the advantage of constraining model parameters so that the effects meet the proportional odds assumption. After finding that the results were similar in both sets of models, and since the proportional odds assumption held for most of the model parameters, we decided to present the results from the ordered logistic regression models in this article. The entire statistical analysis of the study was performed with STATA/MP 13.0 (StataCorp LP, College Station, Texas, United States of America).

## Results

Descriptive characteristics of the study samples by sex are presented in Table 1. One-third of respondents were 60–64 years old, and the distribution of respondents across age groups was similar for both males and females. An important gender difference was revealed in the fact that nine out of ten males were currently married while the same was true for only three out of ten females. The overall literacy rate was 27% and it was higher among males (40%) than females (13%). In addition, 31% of the sample resided in urban areas. In terms of geographic division, the highest percentage of individuals hailed from Dhaka.

**Table 1:** Sample characteristics

	Male (*n* = 2145)	Female (*n* = 2031)	Total (*N* = 4176)
	%	%	%
**Age groups**			
60–64	34.83	35.12	34.96
65–69	23.82	22.11	22.99
70–74	18.46	18.41	18.44
75–79	10.16	9.75	9.96
80+	12.73	14.62	13.65
**Chi-square value (** ***p*** **-value)**	**4.29 (0.37)**		
**Marital status**			
Currently married	91.28	31.41	62.16
Others	8.72	68.59	37.84
**Chi-square value (** ***p*** **-value)**	**1600.00 (0.00)**		
**Education**			
Literate	40.09	13.10	26.96
Illiterate	59.91	86.90	73.04
**Chi-square value (** ***p*** **-value)**	**386.07 (0.00)**		
**Wealth index**			
Poor	40.09	43.62	41.81
Middle	38.79	37.47	38.15
Rich	21.12	18.91	20.04
**Chi-square value (** ***p*** **-value)**	**6.13 (0.05)**		
**Place of residence**			
Urban	31.66	31.12	31.39
Rural	68.34	68.88	68.61
**Chi-square value (** ***p*** **-value)**	**0.14 (0.71)**		
**Division**			
Barisal	10.16	9.85	10.01
Chittagong	20.47	19.25	19.88
Dhaka	27.27	27.97	27.61
Khulna	13.89	13.39	13.65
Rajshahi	10.40	12.56	11.45
Rangpur	9.42	9.06	9.24
Sylhet	8.39	7.93	8.17
**Chi-square value (** ***p*** **-value)**	**5.92 (0.43)**		

The prevalence of having at least one chronic conditions was 38% (see Table 2). Having problems with sight was the most frequently reported disability, followed by disability in walking, hearing, remembering, self-care, and communicating. Thirty-seven percent of respondents were found to have moderate disability in functional domains, and 5% of respondents had severe/extreme disability in functional domains. Four percent of respondents had moderate disability in self-care and 3% had severe/extreme disability in self-care. General disability showed that 38% of respondents had moderate disability and 5% with severe/extreme disability. Gender differences in all disabilities except for disability in remembering were found in Bangladesh. In each disability, elderly females reported moderate, severe, and extreme forms of disabilities in significantly higher percentages than elderly males, with exceptions for severe forms of functional disability and general disability.

**Table 2:** Chronic conditions and disability status of older adults in Bangladesh

	Male (*n* = 2145)	Female (*n* = 2031)	Total (*﻿N*﻿ =﻿ 4176)
	%	%	%
**Suffering from at least one chronic condition**			
No	61.35	62.63	61.97
Yes	38.65	37.37	38.03
**Chi-square value (** ***p*** **-value)**	**0.72 (0.40)**		
**Seeing difficulty**			
Moderate	24.38	31.12	27.66
Severe	4.24	4.58	4.41
Extreme	0.23	0.59	0.41
**Chi-square value (** ***p*** **-value)**	**29.19 (0.00)**		
**Hearing difficulty**			
Moderate	9.00	11.72	10.32
Severe	2.00	2.31	2.16
Extreme	0.19	0.34	0.26
**Chi-square value (** ***p*** **-value)**	**10.15 (0.02)**		
**Walking difficulty**			
Moderate	11.33	14.13	12.69
Severe	3.50	3.35	3.42
Extreme	0.23	0.54	0.38
**Chi-square value (** ***p*** **-value)**	**10.21 (0.02)**		
**Remembering difficulty**			
Moderate	6.25	7.68	6.94
Severe	1.31	1.67	1.48
Extreme	0.19	0.44	0.31
**Chi-square value (** ***p*** **-value)**	**6.74 (0.08)**		
**Self-care difficulty**			
Moderate	2.42	5.61	3.98
Severe	2.05	2.26	2.16
Extreme	0.19	0.59	0.38
**Chi-square value (** ***p*** **-value)**	**32.98 (0.00)**		
**Communication difficulty**			
Moderate	2.47	3.55	2.99
Severe	0.89	1.38	1.13
Extreme	0.09	0.44	0.26
**Chi-square value (** ***p*** **-value)**	**11.52 (0.01)**		
**Functional disability**			
Moderate	33.47	41.70	37.48
Severe	5.13	4.04	4.6
Extreme	0.23	0.34	0.29
**Chi-square value (** ***p*** **-value)**	**31.52 (0.00)**		
**General disability**			
Moderate	33.61	42.10	37.74
Severe	5.03	3.89	4.48
Extreme	0.23	0.30	0.26
**Chi-square value (** ***p*** **-value)**	**33.04 (0.00)**		

Table 3 showed crude as well as adjusted odds ratios of the socio-demographics associated with functional disability by male and female elderly in Bangladesh. The adjusted odds ratios across socio-demographic variables varied by gender, but they were in the same direction for both male and female elderly, except for Khulna and Rangpur divisions. Bivariate associations based on crude odds ratios as well as associations based on adjusted odds ratios from multivariate models showed that older males and females, respectively, had significantly higher odds for functional disability than younger males and females, with statistically insignificant odds for males of 65–69 years. Among males aged 70–74 years, the odds for higher disability were 1.80 times greater than for males aged 60–64 years, while among females aged 70–74 years, the odds for higher disability were 2.11 times greater than for females aged 60–64 years. The odds of having functional disability were greater for elderly females than elderly males. Analyses based on the entire data set that adjusted for sex as an independent variable (see Table 4) confirmed that elderly females were more likely to have functional disability than elderly males. Bivariate associations showed that currently married male and female elderly had lower odds for functional disability than those who were not married, but this association became insignificant in the fully adjusted model. Both males and females suffering from one chronic condition were worse off than those who were free from any chronic conditions in terms of functional disability. This is also observed in Table 4. Female individuals from rich family backgrounds had lower odds for functional disability than female individuals from poor family backgrounds. Individuals from rural areas had higher odds ratios for functional disability than individuals from urban areas, but this was not statistically significant for female elderly. Male elderly from Rajshahi had higher odds ratios compared to male elderly from Barisal division, whereas female elderly from Chittagong, Khulna and Sylhet divisions were found to have lower odds ratios compared to female elderly from Barisal division. More importantly, results for the complete data sets (see Table 4) exerted similar output in terms of direction as Table 3.

**Table 3:** Gender difference in factors affecting functional disability among older adults in Bangladesh

	Male (*N* = 2145)								Female (*N* = 2031)							
	COR	*p*-value	LL	UL	OR	*p*-value	LL	UL	COR	*p*-value	LL	UL	OR	*p*-value	LL	UL
Age groups (reference: 60–64)																
65–69	1.22	0.11	0.95	1.55	1.21	0.14	0.94	1.55	1.30	0.03	1.02	1.65	1.28	0.06	1.00	1.64
70–74	1.92	0.00	1.49	2.47	1.80	0.00	1.39	2.34	2.08	0.00	1.62	2.68	2.11	0.00	1.62	2.75
75–79	2.49	0.00	1.84	3.37	2.43	0.00	1.78	3.32	2.84	0.00	2.08	3.88	3.08	0.00	2.22	4.28
80+	4.06	0.00	3.08	5.36	3.93	0.00	2.95	5.25	3.85	0.00	2.92	5.07	4.01	0.00	2.98	5.38
Marital status (reference: others)																
Currently Married	0.62	0.00	0.47	0.83	0.82	0.19	0.60	1.11	0.67	0.00	0.55	0.81	0.95	0.60	0.77	1.16
Education (reference: literate)																
Illiterate	1.13	0.16	0.95	1.35	1.05	0.65	0.86	1.27	1.16	0.27	0.89	1.50	0.80	0.14	0.60	1.07
Suffering from at least one chronic condition (reference: no)																
Yes	2.22	0.00	1.86	2.64	2.33	0.00	1.94	2.80	2.09	0.00	1.75	2.51	2.22	0.00	1.84	2.68
Wealth index (reference: poor)																
Middle	0.74	0.00	0.61	0.90	0.88	0.24	0.72	1.09	0.82	0.04	0.68	0.99	0.81	0.04	0.66	0.99
Rich	0.68	0.00	0.54	0.86	0.91	0.50	0.69	1.20	0.62	0.00	0.49	0.79	0.58	0.00	0.43	0.77
Residence (reference: urban)																
Rural	1.27	0.01	1.05	1.53	1.28	0.02	1.04	1.58	1.14	0.16	0.95	1.38	1.13	0.25	0.92	1.40
Division (reference: Barisal)																
Chittagong	0.70	0.04	0.49	0.99	0.75	0.12	0.52	1.08	0.55	0.00	0.39	0.78	0.61	0.01	0.42	0.87
Dhaka	1.00	0.98	0.73	1.39	0.97	0.86	0.69	1.36	0.73	0.06	0.53	1.01	0.79	0.16	0.56	1.10
Khulna	1.20	0.32	0.84	1.72	1.28	0.20	0.88	1.86	0.64	0.02	0.45	0.93	0.67	0.04	0.46	0.98
Rajshahi	1.53	0.03	1.05	2.22	1.78	0.00	1.21	2.63	1.03	0.86	0.72	1.49	1.16	0.44	0.80	1.70
Rangpur	1.09	0.66	0.74	1.62	1.13	0.57	0.75	1.70	0.90	0.60	0.60	1.34	1.02	0.92	0.68	1.54
Sylhet	0.76	0.19	0.50	1.15	0.72	0.14	0.47	1.11	0.44	0.00	0.29	0.67	0.50	0.00	0.32	0.77

*Abbreviations*: *COR* Crude odds ratio, *OR* Odds ratio, *LL* Lower limit, *UL* Upper limit

**Table 4:** Factors affecting functional disability among older adults in Bangladesh (*N* = 4176)

	COR	*p*-value	LL	UL	OR	*p*-value	LL	UL
Age groups (reference: 60–64)								
65–69	1.25	0.01	1.05	1.48	1.24	0.02	1.04	1.48
70–74	1.99	0.00	1.67	2.37	1.93	0.00	1.61	2.32
75–79	2.63	0.00	2.12	3.26	2.68	0.00	2.14	3.35
80+	3.95	0.00	3.25	4.80	3.89	0.00	3.17	4.77
Sex (reference: male)								
Female	1.29	0.00	1.15	1.46	1.24	0.01	1.05	1.46
Marital status (reference: others)								
Currently Married	0.65	0.00	0.58	0.74	0.91	0.31	0.77	1.08
Education (reference: literate)								
Illiterate	1.23	0.00	1.07	1.42	0.95	0.51	0.81	1.11
Suffering from at least one chronic condition (reference: no)								
Yes	2.14	0.00	1.89	2.42	2.26	0.00	1.98	2.57
Wealth index (reference: poor)								
Middle	0.77	0.00	0.68	0.89	0.83	0.01	0.72	0.96
Rich	0.65	0.00	0.55	0.76	0.73	0.00	0.60	0.89
Residence (reference: urban)								
Rural	1.21	0.01	1.06	1.38	1.20	0.02	1.03	1.39
Division (reference: Barisal)								
Chittagong	0.62	0.00	0.49	0.79	0.68	0.00	0.53	0.88
Dhaka	0.86	0.20	0.69	1.08	0.88	0.28	0.69	1.11
Khulna	0.89	0.36	0.69	1.15	0.92	0.56	0.71	1.20
Rajshahi	1.27	0.07	0.98	1.65	1.42	0.01	1.08	1.86
Rangpur	0.99	0.95	0.75	1.31	1.06	0.71	0.79	1.41
Sylhet	0.58	0.00	0.43	0.78	0.60	0.00	0.44	0.82

*Abbreviations*: *COR* Crude odds ratio, *OR* Odds ratio, *LL* Lower limit, *UL* Upper limit

Similar to Table 3, Table 5 shows crude as well as adjusted odds ratios of the socio-demographics associated with self-care by male and female elderly in Bangladesh. As found for functional disability, the odds of having self-care disability also increased at older ages. Except for the age group 65–69, female elderly had odds ratios in higher degrees than male elderly. Analyses based on the complete data sets controlling for sex as an independent variable (see Table 6) confirmed that the odds of having self-care disability were greater for elderly females than for elderly males. Both males and females suffering from at least one chronic condition had higher odds of having self-care disability than those who were free from any chronic conditions. The odds of having self-care disability were greater for elderly females from rich families than females from poor families. Both male and female individuals from rural areas had higher odds ratios for self-care disability than their counterparts from urban areas. Results for the pooled data sets (see Table 6) exerted similar output in terms of direction as Table 5. Individuals from rich families also displayed greater odds of having self-care disability than individuals from poor families in the pooled data sets (see Table 6).

**Table 5:** Gender difference in factors affecting self-care among older adults in Bangladesh

	Male (*N* = 2145)								Female (*N* = 2031)							
	COR	*p*-value	LL	UL	OR	*p*-value	LL	UL	COR	*p*-value	LL	UL	OR	*p*-value	LL	UL
Age groups (reference: 60–64)																
65–69	2.66	0.01	1.34	5.31	2.52	0.01	1.26	5.06	1.63	0.12	0.88	3.02	1.66	0.11	0.89	3.10
70–74	2.53	0.01	1.22	5.27	2.28	0.03	1.08	4.80	2.67	0.00	1.49	4.77	2.57	0.00	1.42	4.66
75–79	2.96	0.01	1.31	6.70	2.86	0.01	1.25	6.56	5.37	0.00	2.98	9.68	5.45	0.00	2.96	10.01
80+	8.45	0.00	4.41	16.18	8.13	0.00	4.16	15.88	11.17	0.00	6.73	18.55	10.58	0.00	6.18	18.12
Marital status (reference: others)																
Currently Married	0.57	0.06	0.32	1.03	0.84	0.58	0.45	1.56	0.47	0.00	0.32	0.70	0.89	0.61	0.58	1.38
Education (reference: literate)																
Illiterate	1.15	0.50	0.76	1.75	1.12	0.63	0.71	1.76	1.91	0.03	1.07	3.42	1.39	0.30	0.75	2.60
Suffering from at least one chronic condition (reference: no)																
Yes	2.86	0.00	1.89	4.34	3.05	0.00	1.99	4.69	1.69	0.00	1.23	2.31	1.73	0.00	1.24	2.41
Wealth index (reference: poor)																
Middle	1.15	0.54	0.73	1.80	1.32	0.25	0.82	2.14	0.89	0.52	0.62	1.27	0.95	0.81	0.65	1.40
Rich	1.00	0.99	0.57	1.73	1.29	0.44	0.68	2.43	1.32	0.18	0.88	1.97	1.65	0.04	1.03	2.63
Residence (reference: urban)																
Rural	1.77	0.02	1.08	2.89	1.77	0.03	1.04	2.99	1.90	0.00	1.29	2.80	2.33	0.00	1.51	3.59
Division (reference: Barisal)																
Chittagong	1.11	0.78	0.55	2.23	1.19	0.65	0.57	2.47	0.84	0.52	0.49	1.43	0.95	0.85	0.53	1.68
Dhaka	0.69	0.31	0.34	1.42	0.66	0.28	0.31	1.39	0.62	0.07	0.36	1.04	0.72	0.24	0.41	1.25
Khulna	0.72	0.42	0.32	1.62	0.80	0.60	0.34	1.86	0.37	0.01	0.18	0.74	0.42	0.02	0.20	0.87
Rajshahi	0.87	0.74	0.37	2.01	1.06	0.89	0.45	2.53	0.67	0.20	0.36	1.23	0.78	0.45	0.41	1.49
Rangpur	0.43	0.12	0.15	1.25	0.50	0.21	0.17	1.48	0.55	0.10	0.27	1.11	0.71	0.38	0.34	1.51
Sylhet	0.99	0.98	0.42	2.35	0.86	0.74	0.35	2.11	0.67	0.26	0.33	1.34	0.75	0.43	0.36	1.55

*Abbreviations*: *COR* Crude odds ratio, *OR* Odds ratio, *LL* Lower limit, *UL* Upper limit

**Table 6:** Factors affecting self-care among older adults in Bangladesh (*N* = 4176)

	COR	*p*-value	LL	UL	OR	*p*-value	LL	UL
Age groups (reference: 60–64)								
65–69	2.03	0.00	1.29	3.20	2.04	0.00	1.29	3.24
70–74	2.60	0.00	1.65	4.10	2.48	0.00	1.56	3.95
75–79	4.28	0.00	2.67	6.87	4.32	0.00	2.66	7.02
80+	10.05	0.00	6.75	14.96	9.55	0.00	6.30	14.47
Sex (reference: male)								
Female	1.88	0.00	1.45	2.42	1.68	0.00	1.18	2.40
Marital status (reference: others)								
Currently Married	0.45	0.00	0.35	0.57	0.88	0.46	0.62	1.24
Education (reference: literate)								
Illiterate	1.69	0.00	1.23	2.31	1.18	0.36	0.83	1.69
Suffering from at least one chronic condition (reference: no)								
Yes	2.03	0.00	1.58	2.59	2.13	0.00	1.64	2.75
Wealth index (reference: poor)								
Middle	0.97	0.82	0.73	1.28	1.09	0.56	0.81	1.47
Rich	1.15	0.40	0.83	1.59	1.52	0.03	1.05	2.21
Residence (reference: urban)								
Rural	1.85	0.00	1.37	2.51	2.10	0.00	1.50	2.93
Division (reference: Barisal)								
Chittagong	0.93	0.73	0.61	1.42	1.00	0.99	0.64	1.56
Dhaka	0.65	0.05	0.43	0.99	0.68	0.09	0.44	1.06
Khulna	0.48	0.01	0.29	0.82	0.55	0.03	0.32	0.95
Rajshahi	0.76	0.29	0.47	1.25	0.84	0.49	0.50	1.40
Rangpur	0.51	0.03	0.29	0.92	0.62	0.12	0.34	1.14
Sylhet	0.78	0.36	0.46	1.33	0.78	0.38	0.44	1.36

*Abbreviations*: *COR* Crude odds ratio, *OR* Odds ratio, *LL* Lower limit, *UL* Upper limit

The crude as well as adjusted odds ratios for the factors associated with the third outcome of general disability by male and female (Additional file 1: Table S1) and for the pooled data sets (Additional file 2: Table S2) were also provided. Additional file 2: Table S2 confirmed that elderly females were more likely to have general disability than elderly males. The results of Additional files 1 and 2: Tables S1 and S2 are similar to the findings from Tables 3 and 4, and this may be attributed to the higher prevalence and influence of functional disability over self-care disability among Bangladeshi seniors.

## Discussion

Two important findings were revealed in this study’s in-depth analyses. First, 42% of Bangladeshi older adults, with a higher prevalence among elderly females, had some form of functional disability, including 5% with a severe/extreme form of functional disability. Furthermore, 7% of Bangladeshi older adults, with a higher prevalence among elderly females, had some form of disability in self-care, including 3% of elderly with a severe/extreme form of self-care disability. Second, elderly females had each of the six disabilities in higher percentages (with a borderline significance level [*p* = 0.08] for difficulty remembering) than elderly males. Compared to elderly males, elderly females had higher odds ratios of having functional disability, disability in self-care and general disability. Additionally, older age, suffering from a chronic condition, wealth status, and place of residence including divisional differences were found to be associated with both functional disability and disability in self-care among male and female older adults in Bangladesh.

To this date, no study has specifically focused on disabilities among older adults in Bangladesh. Utilizing data collected in 2003 from working-age people for the World Health Survey (WHS), a study reported that the disability prevalence for the Bangladeshi people aged 40+ years was 26% [34]. In 2005, a study based on data from individuals of all ages reported that the disability prevalence for people aged 51–64 was 13% and for people aged 65+ was 26% [22]. In 2010, in the Bogra district of Bangladesh, the disability prevalence for people aged 55+ was reported to be 25% [13]. The disability prevalence for rural people of age 60+ years was reported to be 26% in 2010 in another study [35]. A study that used the WG’s disability items found that 33% of older Ugandans of age 50 years and above were reported to be disabled in 2010 [36]. These prevalence rates are not directly comparable to the disability prevalence of the current study due to the differences in measures, cut-offs, settings and time period. For example, the WHS used 4 questions on seeing, moving around, concentrating or remembering and self-care, each with 5 response categories. Direct questioning on hearing, speech, vision, physical, and intellectual impairments were used to measure disability in the study by Titumir and Hossain [22]. In low- and middle-income countries, the disability prevalence is particularly high compared to the high-income countries [37], and disability at older ages is an important public health concern [38]. The incidence and development of disability have negative impacts on the lives of elderly people and those who assist the elderly with regular activities. This situation affects thousands of people and, consequently, constitutes a public health problem in society [11]. Bangladesh has ratified the United Nations Convention on the Rights of Persons with Disabilities and enacted the Disability Welfare Act in 2001. The Ministry of Social Welfare, Bangladesh has allocated a special amount toward the Allowance Programme for Insolvent Persons with Disabilities of all ages in its yearly budget since 2005. Although a monthly allowance of 6.25 USD (1 USD = 80 BDT) per person has been allocated to 0.4 million insolvent persons with disabilities since the 2014 fiscal year (2.5 USD in fiscal year 2005), no specific policies regarding older adults with disabilities exist [39]. As the current study revealed that 42% of older Bangladeshi had some form of functional disability and 7% of older Bangladeshi had some form of disability in self-care, programs aimed at reducing functional disability among seniors should be prioritized in Bangladesh. Among functional disabilities, seeing, walking and hearing difficulty were highly prevalent, suggesting that preventative measures could be taken by providing eyeglasses, hearing aids and assistive devices, as well as facilitating the availability of affordable and high quality cataract surgical services.

Compared to elderly males, elderly females reported having higher functional disability, disability in self-care as well as general disability. This is in line with previous studies done in different settings [12–14, 20, 34, 35, 40–43]. A very limited number of studies found no significant gender differences in disability [36, 44]. Gender differences in functional disability and disability in self-care might be explained by early maternal age at first birth, chronic conditions [45], greater female longevity, exposures to domestic violence [46, 47], gender inequalities in nutritional status, marital status and education [43]. Social and health related issues largely contribute to the higher prevalence of disability in women [16]. Higher prevalence of disability among elderly females could also be due to less than adequate care and services for pregnant/delivering mothers, exposures to domestic violence in early life, and the impact of gender-related life conditions. During the reproductive period, mothers encounter some long lasting health problems which remain undisclosed due to cultural reasons. In addition, intimate partner violence was found to be transmitted across generations in Bangladesh [47]. These health problems and domestic violence can cause women to fall sick with greater frequency during reproductive years as well as in later life [12]. Obstetric fistula is today largely confined to the cultures of tropical poverty [48]. This might be one reason for the higher prevalence of disability among elderly females than elderly males in Bangladesh. Patriarchy is also thought to limit women’s advancement, rights, and a cause of lower status of women in Bangladesh. It could deprive women of many necessities including food, nutrition, health care, secure life, a respectable living, mental peace, and an abuse-free life. In turn, females could have poor health as well as disability [12]. In addition, elderly females were reported to suffer from multiple disadvantages resulting from gender biases, widowhood, ill health, social isolation, and poverty when compared to elderly males [49]. Elderly females, particularly widows, who are without living sons or who live alone, have been considered to be particularly at risk of economic destitution, social isolation, poor health, and death in Bangladesh [50]. In our study, a high percentage of elderly females were widows. Due to longer life expectancy among females and differences in age at marriage between brides and grooms, there were higher percentages of elderly widowed females in Bangladesh at the time of this study. This warrants making elderly females a priority group when programs aimed at reducing disability among seniors are implemented in Bangladesh.

Aging is characterized by a progressive loss of physiological integrity, leading to impaired function and higher vulnerability to death [51]. The current study also revealed that disability prevalence increased at older ages. This result has been found in numerous studies [11–13, 20–23, 26, 34–36, 52]. This research underscores the need to prioritize older adults in reducing disability in Bangladesh. Older adults with a chronic condition had higher rates of functional disability and disability in self-care in the current study. This has also been found in other settings [43]. This study thus also underscores the urgency in reducing the impact of chronic conditions in older adults by providing necessary health care services.

As in the current study, wealth inequality, rural-urban disparities and divisional differences in disability among older adults were also found in other studies [12, 13, 22]. Compared to people from poor households, people from middle income and rich households reported suffering lower functional disability, but higher disability in self-care in the current study. In developed countries as well as developing countries including Bangladesh, people from higher socioeconomic status experience less disability [12, 20, 26, 34, 53–56]. The short set of WG’s questions on disability had been previously combined in a study on Bangladesh [12], but for this research were grouped into two categories- functional and self-care disability. In Bangladesh, rich people have more access to health care services than poor people. They use health care services more than the poor [57], and consequently could have reduced functional disabilities. For instance, the rich could have had cataract surgery and bought glasses to relieve their problems with eye-sight. They may use assistive devices for hearing and walking as well. The rich thus have fewer functional disabilities, which should translate into less self-care disability. But this is not the case. One possible reason might be that wealthy individuals exercise less and engage in less physical work than the poor. Particularly, in rich households, women may perform less physical or weight-bearing labor as they have hired help to do so. We also found that functional limitations had not necessarily introduced self-care disability among the Bangladeshi elderly. This could be due to reporting heterogeneity between the poor and the rich; the rich may consider moderate self-care disability as a disability, while the poor may not. Further, cross-tabulations among disability, sex and age groups from the data used in this study demonstrated that a higher proportion of older than younger and females than males reported having self-care disability rather than functional disability. These results should be interpreted in light of the fact that our sample consists of more women than men of age 80 years and over. This may be the primary reason why people from rich households have higher disability in self-care than people from poor households.

Rural elderly reported suffering higher functional, self-care as well as general disability in the current study. Rural elderly were also found to have greater disability and poor health than urban elderly in other studies [22, 24]. The higher rate of disability in rural areas than urban areas might be due to poorer living conditions, less education, poverty, fewer health care services and facilities, which are essential for consideration in reducing disability and formulating new policy. As was the case in other studies [12, 22], divisional differences in disability were also found in the current study, though not in one specific direction. Compared with older adults from Barisal division, the older adults from Chittagong and Sylhet divisions had lower odds of having functional disability and the older adults from Rajshahi division had higher odds of having functional disability. The economic conditions of the divisions might explain the fact; Chittagong is comprised of industrialized areas, Sylhet receives remittances from working abroad, and both are wealthier than Rajshahi. Older adults from Rajshai division may need additional support to reduce functional disability.

### Strengths and limitations

This study has some limitations. First, we were not able to assess causality among the variables used as the data came from a cross-sectional survey. Second, the data are self-reported. This could be a possible source of bias. However, studies have shown that self-reported disability is consistent with medical diagnoses [15]. We advocate caution in explaining the relationship between wealth and disability in self-care, as we only had a few elderly with self-care disability; a data set concentrating on self-care disability is necessary for a deep investigation on this matter. The main strength of this study is that the data came from a large, valid and reliable, nationally representative data set. This study examined two kinds of disability- functional and self-care disability- a fact which also sets it apart from previous studies. However, a longitudinal study is certainly needed to address health and disability transitions, and the causal relationships between variables.

## Conclusions

This study has provided notable results in terms of disability and representativeness for the whole of Bangladesh. In particular, the prevalence of functional disability is higher than self-care disability among older adults in Bangladesh. Also of note, elderly females reported suffering more disability than elderly males. Thus, programs aimed at reducing functional disability among seniors, and elderly females in particular, should be given the highest priority in Bangladesh. In addition, the findings concerning older age, having a chronic condition, wealth status, and place of residence including divisional differences could be used by policy makers and planners to reduce both functional and self-care disability among older adults in Bangladesh.

## Additional files

Additional file 1: Table S1. Factors affecting general disability among male and female older adults in Bangladesh. (DOC 88 kb)

Additional file 2: Table S2. Factors affecting general disability among older adults in Bangladesh (*N* = 4176). (DOC 70 kb)

## Abbreviations

BBS: Bangladesh Bureau of Statistics
HIES: Household Income and Expenditure Survey
RAD: Rapid Assessment of Disability
WG: Washington Group
