# Supplementary material for: Gender differences in functional disability and self-care among seniors in Bangladesh
Source: BMC Geriatr. 2017 Aug 8;17:177. doi: 10.1186/s12877-017-0577-2 (PMC5549315; doi:10.1186/s12877-017-0577-2)
Supplement: Supplementary file 1 — Factors affecting general disability among male and female older adults in Bangladesh. (DOC 88 kb) [file 12877_2017_577_MOESM1_ESM.doc]

**Supplementary information 1**

**Table S1: Factors affecting general disability among male and female older adults in Bangladesh**

|  | **Male (N = 2,145)** | | | | | | | | **Female (N = 2,031)** | | | | | | | |
| --- | --- | --- | --- | --- | --- | --- | --- | --- | --- | --- | --- | --- | --- | --- | --- | --- |
|  | **COR** | **p-value** | **LL** | **UL** | **OR** | **p-value** | **LL** | **UL** | **COR** | **p-value** | **LL** | **UL** | **OR** | **p-value** | **LL** | **UL** |
| **Age groups (reference: 60-64)** |  |  |  |  |  |  |  |  |  |  |  |  |  |  |  |  |
| 65-69 | 1.22 | 0.11 | 0.96 | 1.55 | 1.21 | 0.13 | 0.94 | 1.56 | 1.29 | 0.04 | 1.01 | 1.64 | 1.27 | 0.06 | 0.99 | 1.63 |
| 70-74 | 1.95 | 0.00 | 1.52 | 2.51 | 1.85 | 0.00 | 1.42 | 2.39 | 2.07 | 0.00 | 1.61 | 2.66 | 2.09 | 0.00 | 1.61 | 2.73 |
| 75-79 | 2.51 | 0.00 | 1.85 | 3.39 | 2.45 | 0.00 | 1.80 | 3.34 | 2.83 | 0.00 | 2.07 | 3.87 | 3.06 | 0.00 | 2.20 | 4.24 |
| 80+ | 4.09 | 0.00 | 3.10 | 5.40 | 3.98 | 0.00 | 2.98 | 5.31 | 3.84 | 0.00 | 2.91 | 5.06 | 3.96 | 0.00 | 2.95 | 5.32 |
| **Marital status (reference: others)** |  |  |  |  |  |  |  |  |  |  |  |  |  |  |  |  |
| Currently Married | 0.62 | 0.00 | 0.47 | 0.83 | 0.82 | 0.20 | 0.60 | 1.11 | 0.66 | 0.00 | 0.55 | 0.80 | 0.94 | 0.55 | 0.76 | 1.16 |
| **Education (reference: literate)** |  |  |  |  |  |  |  |  |  |  |  |  |  |  |  |  |
| Illiterate | 1.14 | 0.15 | 0.96 | 1.36 | 1.05 | 0.61 | 0.86 | 1.28 | 1.17 | 0.23 | 0.91 | 1.51 | 0.82 | 0.18 | 0.62 | 1.10 |
| **Suffering from at least one chronic condition** **(reference: no)** |  |  |  |  |  |  |  |  |  |  |  |  |  |  |  |  |
| Yes | 2.20 | 0.00 | 1.84 | 2.62 | 2.31 | 0.00 | 1.93 | 2.78 | 2.08 | 0.00 | 1.74 | 2.49 | 2.20 | 0.00 | 1.83 | 2.65 |
| **Wealth index (reference: poor)** |  |  |  |  |  |  |  |  |  |  |  |  |  |  |  |  |
| Middle | 0.74 | 0.00 | 0.61 | 0.90 | 0.89 | 0.28 | 0.72 | 1.10 | 0.83 | 0.06 | 0.69 | 1.00 | 0.82 | 0.06 | 0.67 | 1.01 |
| Rich | 0.69 | 0.00 | 0.54 | 0.86 | 0.92 | 0.54 | 0.69 | 1.21 | 0.63 | 0.00 | 0.50 | 0.80 | 0.60 | 0.00 | 0.45 | 0.79 |
| **Residence (reference: urban)** |  |  |  |  |  |  |  |  |  |  |  |  |  |  |  |  |
| Rural | 1.27 | 0.01 | 1.05 | 1.53 | 1.29 | 0.02 | 1.04 | 1.59 | 1.15 | 0.14 | 0.96 | 1.39 | 1.14 | 0.21 | 0.93 | 1.41 |
| **Division (reference: Barisal)** |  |  |  |  |  |  |  |  |  |  |  |  |  |  |  |  |
| Chittagong | 0.69 | 0.04 | 0.49 | 0.98 | 0.74 | 0.11 | 0.52 | 1.07 | 0.55 | 0.00 | 0.39 | 0.77 | 0.60 | 0.01 | 0.42 | 0.86 |
| Dhaka | 1.00 | 0.98 | 0.73 | 1.39 | 0.97 | 0.86 | 0.69 | 1.36 | 0.73 | 0.05 | 0.53 | 1.00 | 0.78 | 0.15 | 0.56 | 1.09 |
| Khulna | 1.22 | 0.29 | 0.85 | 1.74 | 1.30 | 0.17 | 0.89 | 1.89 | 0.64 | 0.02 | 0.44 | 0.92 | 0.66 | 0.03 | 0.45 | 0.97 |
| Rajshahi | 1.53 | 0.03 | 1.05 | 2.23 | 1.78 | 0.00 | 1.21 | 2.63 | 1.02 | 0.92 | 0.71 | 1.47 | 1.14 | 0.49 | 0.78 | 1.67 |
| Rangpur | 1.09 | 0.66 | 0.74 | 1.62 | 1.13 | 0.57 | 0.75 | 1.70 | 0.91 | 0.64 | 0.61 | 1.35 | 1.04 | 0.85 | 0.69 | 1.57 |
| Sylhet | 0.76 | 0.19 | 0.50 | 1.15 | 0.72 | 0.14 | 0.47 | 1.11 | 0.43 | 0.00 | 0.28 | 0.66 | 0.49 | 0.00 | 0.31 | 0.76 |

**Notes:** COR = Crude odds ratio; OR = Odds ratio; LL = Lower limit; UL = Upper limit
