# Supplementary material for: Gender differences in functional disability and self-care among seniors in Bangladesh
Source: BMC Geriatr. 2017 Aug 8;17:177. doi: 10.1186/s12877-017-0577-2 (PMC5549315; doi:10.1186/s12877-017-0577-2)
Supplement: Supplementary file 2 — Factors affecting general disability among older adults in Bangladesh (N = 4176). (DOC 70 kb) [file 12877_2017_577_MOESM2_ESM.doc]

**Supplementary information 2**

**Table S2: Factors affecting general disability among older adults in Bangladesh (N = 4,176)**

|  | **COR** | **p-value** | **LL** | **UL** | **OR** | **p-value** | **LL** | **UL** |
| --- | --- | --- | --- | --- | --- | --- | --- | --- |
| **Age groups (reference: 60-64)** |  |  |  |  |  |  |  |  |
| 65-69 | 1.24 | 0.01 | 1.05 | 1.48 | 1.24 | 0.02 | 1.04 | 1.48 |
| 70-74 | 2.00 | 0.00 | 1.68 | 2.39 | 1.94 | 0.00 | 1.62 | 2.33 |
| 75-79 | 2.63 | 0.00 | 2.12 | 3.26 | 2.68 | 0.00 | 2.14 | 3.35 |
| 80+ | 3.96 | 0.00 | 3.26 | 4.81 | 3.89 | 0.00 | 3.17 | 4.78 |
| **Sex (reference: male)** |  |  |  |  |  |  |  |  |
| Female | 1.30 | 0.00 | 1.15 | 1.47 | 1.24 | 0.01 | 1.05 | 1.46 |
| **Marital status (reference: others)** |  |  |  |  |  |  |  |  |
| Currently Married | 0.65 | 0.00 | 0.57 | 0.74 | 0.91 | 0.29 | 0.77 | 1.08 |
| **Education (reference: literate)** |  |  |  |  |  |  |  |  |
| Illiterate | 1.24 | 0.00 | 1.08 | 1.43 | 0.96 | 0.60 | 0.81 | 1.13 |
| **Suffering from at least one chronic condition** **(reference: no)** |  |  |  |  |  |  |  |  |
| Yes | 2.12 | 0.00 | 1.87 | 2.40 | 2.24 | 0.00 | 1.97 | 2.55 |
| **Wealth index (reference: poor)** |  |  |  |  |  |  |  |  |
| Middle | 0.78 | 0.00 | 0.68 | 0.89 | 0.84 | 0.02 | 0.73 | 0.98 |
| Rich | 0.65 | 0.00 | 0.55 | 0.77 | 0.74 | 0.00 | 0.61 | 0.90 |
| **Residence (reference: urban)** |  |  |  |  |  |  |  |  |
| Rural | 1.21 | 0.00 | 1.06 | 1.38 | 1.21 | 0.01 | 1.04 | 1.40 |
| **Division (reference: Barisal)** |  |  |  |  |  |  |  |  |
| Chittagong | 0.62 | 0.00 | 0.48 | 0.79 | 0.67 | 0.00 | 0.52 | 0.87 |
| Dhaka | 0.86 | 0.19 | 0.69 | 1.08 | 0.88 | 0.27 | 0.69 | 1.11 |
| Khulna | 0.89 | 0.37 | 0.69 | 1.15 | 0.93 | 0.58 | 0.71 | 1.21 |
| Rajshahi | 1.26 | 0.08 | 0.97 | 1.64 | 1.41 | 0.01 | 1.08 | 1.85 |
| Rangpur | 1.00 | 0.98 | 0.75 | 1.31 | 1.07 | 0.66 | 0.80 | 1.42 |
| Sylhet | 0.58 | 0.00 | 0.43 | 0.78 | 0.60 | 0.00 | 0.44 | 0.81 |

**Notes:** COR = Crude odds ratio; OR = Odds ratio; LL = Lower limit; UL = Upper limit
